# Effect of tumor microenvironment on pathogenesis of the head and neck squamous cell carcinoma: a systematic review

**DOI:** 10.1186/s12943-019-0983-5

**Published:** 2019-03-30

**Authors:** Barbora Peltanova, Martina Raudenska, Michal Masarik

**Affiliations:** 1 Department of Pathological Physiology, Faculty of Medicine, Masaryk University, Kamenice 5, CZ-625 00 Brno, Czech Republic; 2 Department of Physiology, Faculty of Medicine, Masaryk University, Kamenice 5, CZ-625 00 Brno, Czech Republic; 3 BIOCEV, First Faculty of Medicine, Charles University, Průmyslová 595,, CZ-252 50 Vestec, Czech Republic

**Keywords:** Tumor microenvironment, Head and neck cancer, Tumor metabolism, Epithelial-mesenchymal transition

## Abstract

The tumor microenvironment (TME) is comprised of many different cell populations, such as cancer-associated fibroblasts and various infiltrating immune cells, and non-cell components of extracellular matrix. These crucial parts of the surrounding stroma can function as both positive and negative regulators of all hallmarks of cancer development, including evasion of apoptosis, induction of angiogenesis, deregulation of the energy metabolism, resistance to the immune detection and destruction, and activation of invasion and metastasis. This review represents a summary of recent studies focusing on describing these effects of microenvironment on initiation and progression of the head and neck squamous cell carcinoma, focusing on oral squamous cell carcinoma, since it is becoming clear that an investigation of differences in stromal composition of the head and neck squamous cell carcinoma microenvironment and their impact on cancer development and progression may help better understand the mechanisms behind different responses to therapy and help define possible targets for clinical intervention.

## Introduction

The head and neck cancer (HNC) is considered one of the malignities with the most severe impact on quality of life of patients, caused mainly by relatively low responsiveness to treatment and severe drug resistance [1–3]. HNC is a heterogeneous group of tumors arising from the mucosal surfaces of the nasal and oral cavity, oropharynx, larynx and hypopharynx. Up to 90% of these tumors are head and neck squamous cell carcinomas (HNSCCs) [4], which represent the sixth most prevalent cancer worldwide [5]. The survival rate still remains very low, since up to 25% of patients develop second cancer within 5 years after diagnosis [6]. The most important prognostic determinant of HNSCC tumors is considered the presence of lymph node metastases, since lymphatic metastatic spread correlates with a significant decrease in the survival rate of patients [7]. While primary risk factors are tobacco use and alcohol consumption [8], the role of the oncogenic human papillomaviruses (HPVs) has been implicated in HNSCC as well and many studies have suggested HPV infection as a risk factor of the HNSCC development [9–11].

In recent years, the outlook on cancer has changed dramatically and the tumor is no longer viewed as a bulk of malignant cancer cells, but rather as a complex tumor microenvironment (TME) that other subpopulations of cells corrupted by cancer cells get recruited into to form a self-sufficient biological structure. The stromal component of the tumor microenvironment is composed of multiple different cell types, such as cancer-associated fibroblasts, neutrophils, macrophages, regulatory T cells, myeloid-derived suppressor cells, natural killer cells, platelets and mast cells. These subpopulations of cells interact with each other as well as cancer cells via complex communication networks through various secreted cytokines, chemokines, growth factors and proteins of the extracellular matrix (ECM). This review will focus on describing these major subpopulations of cells and other factors influencing the TME and will discuss their function in the development of cancer, in particular HNSCC.

### Tumor microenvironment

#### Cancer-associated fibroblasts

Cancer-associated fibroblasts (CAFs) are the predominant cell type within the tumor stroma and their main function is to maintain a favorable microenvironment for tumor cell growth and proliferation. CAFs modulate the microenvironment primarily via secretion of large variety of autocrine and paracrine cytokines and other tumor-promoting factors critical for tumor cell proliferation, angiogenesis, invasion, inflammation, metastasis and drug resistance. These factors include various growth factors, cytokines and chemokines, such as epidermal growth factor (EGF), hepatocyte growth factor (HGF), vascular endothelial growth factor (VEGF), C-X-C motif chemokine ligands (CXCCLs) CXCL12 and CXCL14, C-C motif chemokine ligands (CCLs) CCL5 and CCL7, and interleukins (ILs) IL-6 and IL-17A [12–19]. CAFs are also crucial producers of matrix-metalloproteinases (MMPs) and therefore play an important role in modulating the microenvironment by remodelation and degradation of ECM, which ultimately results in promotion of the invasive phenotype of cancer cells [20–22].

The morphology of CAFs is characterized by their elongated spindle-like shape, sharing many similarities with mesenchymal and smooth muscle cells [23]. CAFs have distinctly different morphological and biological characteristics compared with normal fibroblasts; they also differ from normal fibroblasts by their constitutively activated state. Several molecules, such as α-smooth muscle actin (α-SMA), fibroblast activation protein (FAP), fibroblast-specific protein-1 (FSP-1), platelet-derived growth factor receptor α/β (PDGFR α/β) and vimentin are considered some of the markers of activated CAFs [12, 24–26].

CAFs can be derived from various types of progenitor cells, such as resting resident fibroblasts or pericytes through mesothelial-mesenchymal transition (MMT) [27], endothelial cells through endothelial-mesenchymal transition (EdMT) [28], epithelial cells through epithelial-mesenchymal transition (EMT) [29], adipocytes [30] and bone marrow-derived mesenchymal cells (BDMCs) [31]. The most common marker used to detect CAFs in the tumor stroma is α-SMA, a specific marker of myofibroblasts [32]. This myofibroblast phenotype of CAFs is frequently observed in HNSCC and the upregulation of α-SMA has been correlated to poor prognosis in oral carcinoma [33]. Another marker of myofibroblasts widely used for detection of CAFs is FAP [34, 35]. FAP is overexpressed in sites of fibrosis and in the tumor stroma of various carcinomas, including HNSCC. CAFs can also be characterized by the absence of epithelial and endothelial markers, such as cluster of differentiation (CD) CD31 and cytokeratin [36, 37].

#### Macrophages

Macrophages are mononuclear phagocytes considered one of the most important immune cells, mainly for their prominent active role in tissue homeostasis and both innate and acquired immune response against pathogens [38]. Macrophages display a great plasticity, M1 and M2 representing the extreme activation states. However, the re-polarization of fully polarized macrophages *in vitro* towards the other phenotype by various cytokines has been observed [39]. These two distinct phenotypes are characterized by different receptor expression, function and cytokine and chemokine production [40–43]. The “pro-inflammatory” classically activated M1 macrophages are characterized by their activation by the T helper type 1 (Th1) cytokine interferon-γ (IFN-γ) and/or bacterial lipopolysaccharide (LPS). They produce pro-inflammatory cytokines, such as IL-12, IL-23 and tumor necrosis factor-α (TNF-α), and chemokines (CCL-5, CXCL9, CXCL10 and CXCL5). They participate in anti-tumor immunity by contributing to the Th1 response to infection, by inhibiting proliferation and by exerting cytotoxic activity [44–46]. The “anti-inflammatory” alternatively activated M2 macrophages play an immunoregulatory role and are involved in the tissue remodeling, wound healing, angiogenesis and tumor progression [47–50]. The M2 phenotype is induced by various Th cytokines (IL-4, IL-10, IL-13) and is characterized by increased secretion of anti-inflammatory cytokines, such as IL-1 receptor antagonist (IL-1ra), IL-10 and TGF-β [51–53].

Tumor-associated macrophages (TAMs) represent a major component of the macrophage population largely contributing to proliferation, invasion and metastasis of tumor cells, promotion of tumor progression, angiogenesis and suppression of T cell antitumor immune response. Recent studies suggested the correlation between the level of infiltration of TAMs and a poor outcome in HNSCC, which could be used as a potential prognostic marker [54–56]. In the past years, TAMs have been considered a large subpopulation of macrophages within the M2 phenotype, however it has become clear TAMs are able to adopt a wide range of different activation states between M1 and M2, expressing both M2 and M1 markers, such as upregulated IL-10 (M2) [57], arginase-1 (M2) [58], peroxisome proliferator-activated receptor *γ* (PPAR*γ*) (M2) [59], TNF-α (M1) [60], MMP-9 (M1) [61] and increased levels of interferon-(INF)-inducible chemokines CCL2, CCL5, CXCL9, CXCL10 and CXCL16 (M1) [62].

#### Neutrophils

Neutrophils, also known as polymorphonuclear leukocytes (PMNs), are essential effector cells of the innate immune system and the most predominant leukocyte population present in the circulation [63]. Neutrophils, along with macrophages, represent the first line of defense against pathogens and first responders at the site of infection and injury [64], they are also directly involved in adaptive immunity responses, playing an important role in mediation of T cell independent antibody responses [65], as well as antigen presentation and T cell activation [66, 67]. Until recently, neutrophils were thought to act only as phagocytic cells by producing lytic enzymes and reactive oxygen species (ROS). However, neutrophils are able to form neutrophil extracellular traps (NETs) by releasing their cytotoxic cytosolic and granule proteins on a scaffold of decondensed chromatin [68] in a cell death process called NETosis [69, 70]. It has been reported NETs activate platelets and promote thrombosis [71, 72], and indeed an increased risk of cancer-associated venous thromboembolism (VTE) has been reported in many types of cancer, including the HNSCC [73].

The identification and characterization of the neutrophil population based on the expression of specific surface markers remains difficult since these specific markers have yet to be identified. For the identification of pure human neutrophil subpopulations, many studies use various markers individually or in combination, such as CD11b, CD14, CD15, CD16, CD62L and CD66b [74–76].

The contribution of tumor-associated neutrophils (TANs) to the cancer progression remains unclear, the main reason being TANs show both pro- and anti-tumor properties. In TANs, in an analogy to TAMs, a phenotypic duplicity in a form of polarization states has been observed [77]. These anti-tumor and pro-tumor phenotypes within the neutrophil population have been termed N1 and N2, respectively. The pro-tumor N2 phenotype is characterized by increased expression of angiogenesis and invasion promoting factors CXCR4, VEGF and MMP-9 with absent IFN-β [78] and is acquired by neutrophils following the TGF-β treatment [77]. However, neutrophils can revert back to the cytotoxic N1 phenotype upon the TGF-β blockade or in the presence of the IFN-β [79], while expressing high levels of intercellular adhesion molecule 1 (ICAM1) and TNF-α as well as increasing NETs formation.

#### Myeloid-derived suppressor cells

Myeloid-derived suppressor cells (MDSCs) comprise a heterogeneous population of immature inhibitory immune cells in various stages of myelopoiesis [80]. This cell population plays a crucial role in negative regulation of the immune response in many pathological conditions, such as cancer and inflammation, by inhibiting both the adaptive and innate immunity. MDSCs are induced by various tumor-derived factors in the microenvironment, mainly granulocyte-macrophage colony-stimulating factor (GM-CSF), VEGF and IL-6 [81], and modulate the inflammatory microenvironment via depletion of many amino acids (such as L-arginin, L-tryptophan and L-cystein) [82–84], via increased production of nitric oxid (NO), ROS, inducible NO synthase (iNOS) and arginase-1 [85–87], and via expression of programmed death receptor ligand 1 (PD-L1), which ultimately inhibits T cell activation and proliferation and causes T cell apoptosis [88]. MDSCs also regulate the activity of natural killer (NK) cells and the induction of immunosuppressive regulatory T cells (Tregs) [89, 90].

MDSCs were originally described in peripheral blood of HNSCC patients as immature CD34^+^ cells exhibiting the ability to suppress the activity of T cells [91–93]. The identification of MDSCs based on the expression of surface markers is challenging mainly because of the phenotypic diversity of the MDSCs population, since different subpopulations within the MDSCs express combinations of various myeloid markers, including CD11b, CD33, CD14, CD15 and CD16 but lack the expression of HLA-DR. Although MDSCs have been first discovered for their immune-suppressive function in cancer, recently the presence of MDSCs has also been linked to other processes within the TME, such as promotion of tumor angiogenesis via production of pro-angiogenic factors [94, 95], degradation of ECM via production of significant levels of MMPs, especially MMP-9, and most importantly the formation of premetastatic niches.

#### Regulatory T-cells (Tregs)

Regulatory T cells comprise a unique subset of T cells responsible for suppression of excessive immune response, for maintaining self-tolerance and homeostasis, and for regulation of other immune cells, including CD4 and CD8 T-cells, B cells, NK cells, macrophages and dendritic cells; and the loss of these cells ultimately results in various autoimmune diseases [96]. Tregs are characterized by their expression of markers CD4, CD25 and transcription factor forkhead box P3 (FOXP3) [97]. However, the markers CD4 and CD25 are also expressed by effector T cells, thereby making it difficult to distinguish these two populations. In addition, the intracellular localization of FOXP3 requires cell permeabilization for its detection, which makes the isolation of viable Tregs challenging. Tregs also express high levels of cytotoxic T-lymphocyte-associated protein 4 (CTLA-4) and glucocorticoid-induced tumor necrosis factor receptor family-related protein (GITR) [98, 99].

Treg cells display great heterogeneity within the population, thus can be divided into phenotypically and functionally distinct subpopulations based on their localization, origin and expression profile of markers [100]. CD25^+^CD4^+^ Tregs arising in the thymus, termed natural regulatory T cells, express the FOXP3 transcription factor constitutively and are crucial for the maintenance of self-tolerance. In contrast, peripheral CD25^+^CD4^+^ Tregs can differentiate from conventional mature CD4^+^ T cells outside of the thymus, thus are called induced or adaptive Tregs. These T cells require activation in the presence of cytokines, such as IL-2 and TGF-β, to upregulate FOXP3 [101] and their main function is to prevent local inflammation.

Since their discovery, the molecular mechanisms by which Tregs exert their suppressor function have been intensely studied. It has been observed Tregs can influence the immune system via either contact-dependent or contact-independent mechanisms. Vignali et al. arranged these mechanisms into four modes of action: (1) suppression by inhibitory cytokines (such as IL-10, IL-35 and TGF-β), (2) suppression by cytolysis via granzyme-A/B-dependent and perforin-dependent killing of target cells, (3) suppression of effector T cells by metabolic disruption via depletion of IL-2, and (4) suppression by modulation of dendritic-cell (DC) maturation or function [102].

#### Platelets

Platelets, also known as thrombocytes, are anucleated cells arising as fragments of megakaryocytes in the bone marrow, that serve as another major cellular group of first responders at the site of injury. It has been thought the primary function of platelets is thrombosis, wound healing and maintaining of homeostasis, but in recent years numerous studies started to focus on the role of blood platelets in regard of cancerogenesis, tumor biology and inflammation.

Platelets mediate the tumor microenvironment via three types of secretory granules - dense granules, lysosomes and α-granules. During platelet activation, the cargo from these granules is released into the extracellular environment, leading to platelet aggregation, vasoconstriction and regulation of cell proliferation through secretion of numerous growth factors [103]. The dense granules contain mainly small molecules, including ADP, ATP, calcium, 5-HT (5-hydroxytryptamine, also known as serotonin) and pyrophosphate [104–106]. Dense granules also contain membrane proteins CD63 and lysosomal-associated membrane protein 1/2 (LAMP1/2), glycoprotein-(GP)-Ib, P-selectin, and integrin αII-β3 [107]. Lysosomes represent another type of platelet granules. The function of these granules has not yet been fully eluciated, however they contain an acidic pH with acid hydrolases, which are able to degrade and remodel the ECM and vasculature. Also similarly to dense granules, lysosomes express membrane proteins CD63 and LAMP1/2 [108]. The most abundant group, α-granules, contains a vast number of proteins and factors important in hemostasis, thrombosis and adhesion, including vitronectin, thrombospondin, fibrinogen, fibronectin and von Willebrand factor (VWF). In addition, α-granules contain proteins involved in inflammation and angiogenesis, many mitogenic growth factors, a variety of chemokines and various MMPs [109–113]. The release of these factors from α-granules attracts other cells to form tumor cell–platelet emboli, stimulating tumor cell growth and angiogenesis. α-granules also express number of transmembrane proteins, such as integrins, GP αIIbβ3, CD36, glucose transporter 3 (GLUT3), GPVI and P-selectin [114–117]. P-selectin, a surface protein translocated during platelet activation, is responsible for mediating platelet-leukocyte interactions via binding to leukocyte P-selectin glycoprotein ligand-1 (PSGL-1) [118].

#### Mast cells

Mast cells (MCs) represent another important myeloid component of the immune system that contributes to both innate and acquired immune responses. Like other immune cells, mast cells originate from pluripotent progenitor cells in the bone marrow, which they exit undifferentiated and migrate to target peripheral tissues to complete maturation. This terminal differentiation is strongly regulated by various factors provided by the microenvironment, including stem-cell factor (SCF) and IL-3 [119]. The activation of a mast cell is mediated by the cross-linkage of the IgE receptor (FcεRI) expressed on their surface, which leads to the release of the granule inflammatory cargo into the extracellular space, including histamine, TNF-α, heparin, chondroitin sulfate E, prostaglandin D_2_ (PGD_2_), tryptase, chymase, cathepsin G, carboxypeptidase A (CPA1), leukotriene C_4_ (LTC_4_), various interleukins and GM-CSF [120]. In addition to the rapid secretion of the granule content through exocytosis, mast cells release their contents selectively via piecemeal degranulation [121]. Interestingly, piecemeal degranulation has been particularly detected in areas of chronic inflammation or tumors and has been reported to be a preferred secretory pathway of tumour-associated mast cells (TAMCs) [122].

The aforementioned profile of mediators secreted by TAMCs suggests that TAMCs can play both pro- and anti-tumorigenic roles in cancer development. Tumor-promoting functions of TAMCs include angiogenesis through the production of VEGF and fibroblast growth factor-(FGF)-2 [123], ECM degradation via production of MMPs and various proteases, which results in tumor cell invasion and migration [124], and induction of tumor cell proliferation via production of histamine [125]. In addition, mast cells produce a variety of chemotactic factors in order to recruit other immune cells into the tumor [126, 127]. In contrast, in some types of tumors, the tumor suppressive effects of TAMCs have been reported, mainly by supporting tumor rejection [128] and mediating tumor cell apoptosis via the production of IL-4 and TNF-α [129, 130].

#### Natural killer cells

Natural killer cells (NK cells) play a crucial role in the innate immune system, since their main function in the organism is the ability to quickly detect and kill virus-infected or malignant cells. NK cells are characterized as large granular CD3^-^ lymphocytes that can be classified into two subsets, depending on their expression levels of surface markers CD16 and CD56. CD56^dim^/CD16^bright^ subpopulation constitutes the majority, approximately 90% of all peripheral blood NK cells, and is responsible for high natural cytotoxicity [131]. CD56^bright^/CD16^dim^ subpopulation is characterized by higher expression levels of variety of immunomodulatory cytokines. The most prominent cytokines secreted by NK cells are IFN-γ and TNF-α. However, NK cells have been reported to produce a variety of other important factors, including GM-SCF, IL-5, IL-8, IL-10, IL-13, CCL2, CCL3, CCL4, CCL5 and CXCL10 [132–135].

NK cell function is tightly regulated by the ratio of signals from two different types of receptors present on the cell surface – activating and inhibitory receptors. The self-MHC class I molecules expressed on healthy cells act as inhibitory stimuli preventing NK cell activation [136]. Malignant or virus-infected cells downregulate MHC-I expression in order to escape cytotoxic T cells; this however, results in recognition by NK cells. In addition, activating receptors on the target cells’ surface are upregulated in response to the virus infection or their malignant transformation [137]. The activation of NK cells is then followed by number of possible inductions of apoptosis of target cell, including exocytosis of perforin and granzymes, Fas ligand (FasL), TNF-related apoptosis-inducing ligand (TRAIL) activation or antibody-dependent cellular cytotoxicity (ADCC) [138–141].

In contrast to cytotoxic T cells, NK cells do not require prior sensitization or stimulation for their effector function. However, some recent studies provide evidence that a subpopulation of NK-like cells, termed natural killer T cells (NKT cells), may play an important role in the immune response, since this subpopulation lies at the interface between innate and adaptive immune systems [142]. NKT cells are of lymphoid lineage and they share many morphological and functional characteristics of T cells and NK cells since they are defined by the expression of both T cell and NK cell surface markers [143]. NKT cells require prior priming for their function and can develop antigen-specific immunological memory [144–146]. One subset of NKT cells, the invariant natural killer T cells (iNKT cells), express a highly restricted invariant aβ T cell receptor (TCR) and low levels of these iNKT cells in peripheral blood predict poor outcome in HNSCC patients [147, 148].

These and other aforementioned subpopulations are summarized in Table 1.

**Table 1:** Different cell populations exhibit distinct functions within the tumor microenvironment

Cell type	Markers (human)	Increased production	Activity	Function	Ref.
M1 TAMs	CD68^+^	IL-12, IL-23, TNF-α, CCL-5, CXCL9, CXCL10, CXCL5	anti-tumor	contribution to the Th1 response, inhibition of proliferation, cytotoxic activity	[44–46]
M2 TAMs	CD68^+^	IL-1ra, IL-10, TGF-β, arginase-1	pro-tumor	promotion of tumor progression, angiogenesis, suppression of T cell antitumor immune response	[47–53]
N1 TANs	CD11b^+^, CD14^+^, CD15^+^, CD16^+^, CD62L^+^, CD66b^+^	ICAM1, TNF-α	anti-tumor	cytotoxic activity, increased NET formation	[79]
N2 TANs	CD11b^+^, CD14^+^, CD15^+^, CD16^+^, CD62L^+^, CD66b^+^	CXCR4, VEGF, MMP-9	pro-tumor	promotion of angiogenesis, invasion	[77, 78]
MCs	CD117^+^, CD203c^+^, FcεRI^+^	histamine, heparin, chondroitin sulfate E, PGD_2_, tryptase, chymase, CPA1, LTC_4_, GM-CSF, MMPs, IL-4, TNF-α, cathepsin G	pro-tumor	promotion of angiogenesis, ECM degradation, stimulation of cancer cell proliferation, recruitment of immune cells	[120, 123–127]
MDSCs	CD11b^+^, CD33^+^, CD14^+^, CD15^+^, CD16^+^, HLA-DR^-^	NO, ROS, iNOS, arginase-1, PD-L1, MMP-9	pro-tumor	immunosuppression, inhibition of T cell activation and proliferation, promotion of angiogenesis, degradation of ECM	[82–88, 94, 95]
NK cells	CD3^-^, CD16^+^, CD56^+^	IFN-γ, TNF-α, GM-SCF, IL-5, IL-8, IL-10, IL-13, CCL2, CCL3, CCL4, CCL5, CXCL10	anti-tumor	cytotoxic activity without prior antigen presentation, modulation of adaptive immune response	[132–135, 138–141]
NKT cells	CD3^+^,CD56^+^, CD161^+^, CD1a^+^, CD16^+^	IFN-γ, TNF-α, GM-CSF, TGF-β, IL-2 , IL-4, IL-5, IL-6, IL-10, IL-13, IL-17A	anti-tumor	cytotoxic activity, antigen-specific immunological memory	[142–146]
Tregs	CD4^+^, CD25^+^, FOXP3^+^	IL-10, IL-35, TGF-β, VEGF	pro-tumor	immunosuppression, promotion of angiogenesis	[97–99, 101, 102]
Platelets	CD41^+^, CD42a^+^, CD42b^+^, CD61^+^	ADP, ATP, calcium, 5-HT, CD63, LAMP1/2, GP-Ib, P-selectin, integrin αII-β3, fibrinogen, vitronectin, thrombospondin, fibronectin, VWF, MMPs, GLUT3	pro-tumor	thrombosis, wound healing, maintaining of homeostasis, vasoconstriction, promotion of cell proliferation, immunoevasion by platelet aggregation	[105–107, 109–111, 113–116]
CAFs	α-SMA^+^, FAP^+^, FSP-1^+^, CD33^-^, absent cytokeratin	EGF, HGF, VEGF, CXCL12, CXCL14, CCL5, CCL7, IL-6, IL-17A, MMPs	pro-tumor	stimulation of tumor growth, invasion, angiogenesis, metastasis, induction of chemo- and radio-resistance, ECM degradation	[12–19]

Abbreviations: *TAMs* tumor-associated macrophages, *TANs* tumor-associated neutrophils, *MCs* mast cells, *MDSCs* myeloid-derived suppressor cells, *NK* natural killer cells, *NKT* natural killer T cells, Tregs regulatory T cells, CAFs cancer-associated fibroblasts

#### Extracellular matrix

The extracellular matrix (ECM) is a non-cellular network of macromolecules, including fibrous structural proteins, glycoproteins, growth factors and proteoglycans that form a structure providing other surrounding cells with physical and biochemical support. In cancer, ECM becomes frequently deregulated and disorganized, which directly stimulates malignant cell transformation [149, 150]. ECM produces high amounts of MMPs. MMPs are a group of zinc-dependent protein and peptide hydrolases secreted and activated by malignant cells, capable of degradation of ECM proteins of the basement membrane, as well as other important molecules, such as growth factors, cell surface receptors and adhesion molecules [151–155].

The first hypothesis surrounding the function of MPPs has been attributed to their capability of degrading ECM and helping tumor cells migrate to local and distant sites. In recent years, it has been observed the crucial function of MMPs in the ECM is activating growth factors or releasing them from the matrix, thus promoting the initiation and proliferation of primary tumors. MMPs are also involved in tumor angiogenesis by activating basic fibroblasts growth factor (bFGF), VEGF and TGF-β [156–158]. Although tumor cells were considered to be the source of MMPs in the stroma to help degrade the surrounding ECM, it is now becoming clear that most of the MMPs are produced by the stromal cells in the tumor microenvironment, such as fibroblasts and inflammatory cells [159, 160].

Proteins of ECM, such as collagen, elastin, fibronectin, laminin and tenascin influence cell adhesion and proliferation as well as provide a structural support along which cells migrate out of and into the TME. Increased production of collagen, laminin and elastin also results in elevated stiffness of tumor compared to surrounding normal tissue [161–163]. Increased tumor stiffness has a strong impact on cancer progression by activating oncogenic intracellular signaling, such as Akt, β-catenin, focal adhesion kinase (FAK) and phosphatidylinositol 3-kinase (PI3K) pathways, while simultaneously inhibiting tumor suppressor genes for phosphatase and tensin homolog (PTEN) and glycogen synthase kinase 3α/β (GSK3α/β) [164]. Increased matrix stiffness also promotes the activation of surrounding fibroblasts to a CAF phenotype, which is maintained via mechanosensitive transcription factor yes-associated protein (YAP) [165].

Up to the 30% of the ECM protein mass constitutes of collagen, which provides the cell with tensile strength and support for migration, therefore playing an important role in the regulation of the cell behavior and development [166]. Besides the mechanical and structural contributions, collagens also play a crucial role in a wide range of biological functions, such as tissue scaffolding, cell adhesion, cell differentiation, cell migration and wound repair [167–170]. Along with collagen, one of the most abundant glycoproteins of the ECM is fibronectin (Fn), which is produced by various different cell types, such as fibroblasts and endothelial cells [171, 172]. Fibronectin structure contains binding and interaction sites for several other molecules present in the ECM, such as integrins, fibrin, heparin, tenascin, collagen, gelatin and syndecan [173–177]. In regards to cancer development, increased levels of fibronectin have been associated with tumor progression, migration, invasion and reduced responsiveness to treatment [178–182]. Moreover, CAF-derived matrices exhibit aligned fibronectin organization, which mediates directional migration of cancer cells [183].

#### Metabolic reprogramming of TME

A common feature of the rapid progression of solid tumors is intratumoral hypoxia, which arises as a consequence of insufficient oxygen supply to the tissue. Rapidly growing tumors quickly exhaust the available oxygen, which stimulates an upregulation of production of pro-angiogenic factors, such as VEGF, to form new vessels. However, these newly formed blood vessels are often characteristic of high leakage and irregular structure, which impair their function [184]. Hypoxic microenvironment has also been implicated as a crucial contributor to radio- and multidrug-resistance [185, 186]. Hypoxia leads to upregulation of hypoxia-inducible factor 1 (HIF-1) [187]. HIF-1 represents a key player in mediating the adaptive cellular response to low oxygen levels in the microenvironment. As a major transcription factor, HIF-1 has been implicated in the regulation of the expression of various genes associated with tumor cell growth, survival and proliferation [188–190], including genes involved in cellular energy metabolism. HIF-1 induces upregulation of many glucose transporters (GLUTs) and enzymes (such as lactate dehydrogenase A) [191], thus triggering the shift from oxidative phosphorylation (OXPHOS) to less energetically efficient glycolytic pathway in tumor cells, a process known as the Warburg effect.

Warburg effect describes an observation, in which glucose taken up by the tumor tends to get metabolized into lactate to generate ATP even in a sufficient presence of oxygen via aerobic glycolysis instead of oxidative phosphorylation [192]. It has been suggested, that the Warburg effect may promote the creation of more advantageous TME for cancer cell proliferation, survival and invasion. Due to these metabolic alterations, tumor cells produce elevated amounts of lactate, H^+^ and CO_2_, which results in enhanced acidification of the TME, thus increasing the tumor metastatic potential and resistance to treatment [193–195]. Interestingly, tumor-derived lactate has been reported to contribute to the polarization of TAMs into the M2 phenotype [196]. In addition to glucose, tumor cells can utilize L-lactate as an alternative energy source via lactate shuttle, which is regulated by the conversion of lactate into pyruvate by the lactate dehydrogenase (LDH) as well as by the transport of lactate across the tumor cell plasma membrane [197, 198]. The proton-linked transport of L-lactate, pyruvate, acetate and ketone bodies across the plasma membrane is facilitated by monocarboxylic acid transporters MCT1-MCT4 [199]. In tumors, the influx and efflux of excessive levels of L-lactate into and out of tumor cells are directed by MCT1 and MCT4. The overexpression of these two MCTs has been reported in several tumors, including HNSCC, and has been associated with poor prognosis [200–203]. Many types of cancer, including HNSCC, exhibit a metabolic symbiosis between tumor cells and surrounding stroma, CAFs in particular. A recent study demonstrated that the glycolytic switch in HNSCC cancer cells is induced by CAF-derived HGF and in turn HNSCC-secreted bFGF promotes lactate consumption by CAFs [204].

### TME in the pathogenesis of HNSCC

#### Premalignant lesion

HNSCC is associated with severe immunosuppression, however, the milieu of the premalignant lesion has yet to be well defined. It has been reported that oral leukoplakia shows a significant infiltration of proinflammatory immune cells, such as TAMs, CD8^+^ T cells and NK cells [205–207]. Costa et al. conducted a study to examine the differences in the immunological phenotype of the premalignant and malignant stages of HNSCC using a mouse model of 4-nitroquinoline 1-oxide (4-NQO)-induced oral carcinogenesis [208]. It was observed that the premalignant stage is associated with elevated levels of inflammatory Th1, Tc1 and Th17 cells compared to controls and HNSCC-bearing mice, while the number of Tregs increased in HNSCC-bearing mice. The same mouse model was utilized to investigate the shift in the inflammatory cytokine profile depending on the malignant progression [209]. It has been observed, that premalignant oral lesions are associated with an increased level of IL-17, as well as IL-23, compared to controls or HNSCC, thus promoting the Th17 phenotype. In contrast, HNSCC tissues showed a downregulation of IL-23 and upregulation of TGF-β, most likely to skew the Th17 phenotype toward the Treg phenotype. Another study showed that premalignant lesions secrete many proinflammatory mediators, such as CCL5 (also known as RANTES), monocyte chemoattractant protein 1 (MCP-1), granulocyte-colony stimulating factor (G-CSF) and prostaglandin-E2 (PGE2) compared to HNSCC cells, suggesting the premalignant microenvironment to be more immune stimulatory than the microenvironment of an established HNSCC [210]. Some research has also been conducted on saliva samples of patients with premalignant oral lesions, which showed increased levels of proinflammatory cytokines TNF-α and IL-6 [211–213]. Several studies investigated the effect of immune cell infiltration on the progression of the premalignant lesion to malignant phenotype through angiogenesis. Immunohistochemical analyses have shown the total number of immune cells infiltration to be significantly elevated depending on the severity of the lesion, with lowest numbers observed in normal gingival tissue. In addition, the mast cell density (MCD) significantly correlated with microvessel density (MVD) depending on the progression of the malignancy [214–217].

In addition to the immune cell infiltration, the contribution of CAFs to the progression from the premalignant lesion to oral squamous cell carcinoma (OSCC) has been investigated. These studies have focused on the distribution of the CAFs marker α-SMA, which has been detected in samples of premalignant lesions, while absent in normal epithelium [218–220]. Interestingly, increased frequency of CAFs correlated with the progression from normal mucosa and potentially malignant disorders to an invasive phenotype. Potentially malignant oral leukoplakia also shows elevated expression of ECM components tenascin, MMP-2, as well as FGF-2 and its receptors FGFR-2 and FGFR-3, which are predictive of progression to OSCC [221–223].

#### Primary tumor

**Fig. 1:**
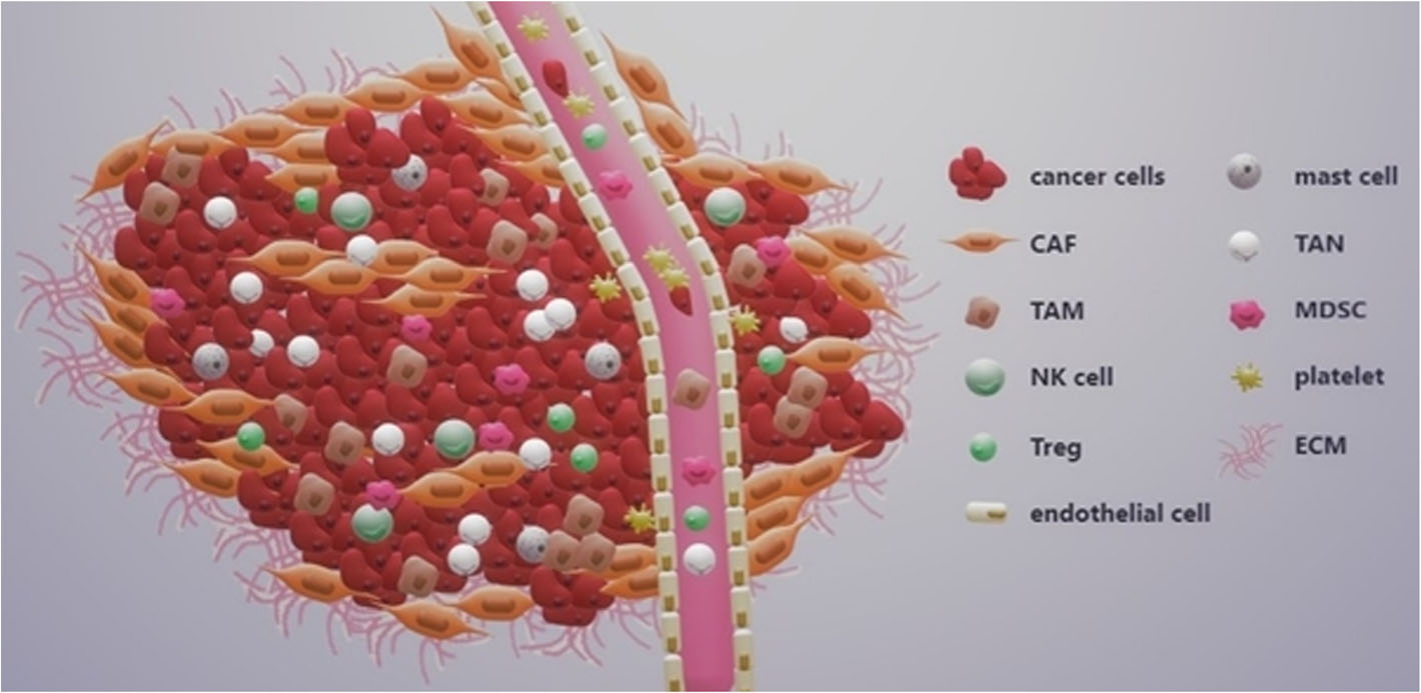
Cellular constituents within the tumor microenvironment. In addition to the cancer cells, the tumor stroma is comprised of many other supporting cell populations as well as the extracellular matrix, which crucially contribute to the tumor progression. The characteristics and function of individual cell populations are described in the Table 1. Abbreviations: TAM tumor-associated macrophage, TAN tumor-associated neutrophil, MDSC myeloid-derived suppressor cell, NK natural killer cell, Treg regulatory T cell, CAF cancer-associated fibroblast, ECM extracellular matrix

The growth of primary tumor is associated with the presence of immune cells, which cause inflammation frequently observed in HNSCC (shown in Fig. 1). Several studies investigated the significance of the overall population of tumor-infiltrating lymphocytes (TILs) as a prognostic marker of HNSCC. In these studies, various representative subsets of TILs, such as CD8+ cytotoxic T cells, CD4+ helper T cells, CD68+ macrophages and MDSCs, CD163+ macrophages, CD57+ NK cells and FOXP3+ Tregs, were evaluated and correlated with clinicopathologic characteristics of HNSCC patients. Immunohistochemical analysis revealed that tumors heavily infiltrated by TILs were associated with better outcome [224–227].

The infiltration of TAMs is a major contributor to the inflammation in HNSCC and is associated with poor prognosis, lymph node metastasis and low survival [228–232]. Kross et al. found the level of monocyte-derived IL-6 predicted recurrence and survival of HNSCC patients using an in vitro coculture system of monocytes with spheroids derived from HNSCC patients [233]. Costa et al. reported a predominance of M2 macrophages expressing TGF-β and IL-10 in oral squamous cell carcinoma (OSCC) group compared with healthy controls, which was further correlated with worse prognosis [234]. A recent study by Jiang et al. showed that compared to peritumoral macrophages OSCC-derived TAMs expressed higher levels of PD-L1, which correlated with increased T cell apoptosis [235], and this has been confirmed by other studies [236]. Beside tumor cells, macrophages also constitute an important source of VEGF, thus may contribute to tumor development via neovascularization [237–240]. Several studies also evaluated the prognostic significance of CD68+ macrophage infiltration regarding HPV status of HNSCC, which show that higher macrophage infiltration in HPV+ compared with HPV- HNSCC correlated with better prognosis [56, 241]. Also, high infiltration of neutrophils in OSCC is associated with poor clinical outcomes. A study by Trellakis et al. showed that high neutrophil infiltration correlated with poor patient survival [242]. This was confirmed by Wang et al., who correlated high neutrophil infiltration with high tumor stage, recurrence and lymph node metastases [243]. An in vitro study by Trellakis et al. investigated the interaction of neutrophils and HNSCC cancer cells, which reported that HNSCC-conditioned medium reduced neutrophil apoptosis, increased chemotaxis of neutrophils and induced the production of MMP-9 and CCL4 by neutrophils [244]. Mast cells influence the primary tumor mainly by the production of many pro-angiogenic factors, such as VEGF, bFGF, TGF, TNF-α, tryptase, heparin and various MMPs, which are associated with ECM degradation, angiogenesis, progression and growth of OSCC [245, 246]. Mast cell and microvessel densities are increased in OSCC compared with normal mucosa, however, no significant correlation has been found [247–251]. Various studies focused on the presence of NK cells in HNSCC patients, in which an increased number of NK cells predicted improved survival [252, 253]. Korrer et al. found that NK cells derived from HNSCC primary tumors significantly downregulated activating receptors NKG2D, DNAM-1, NKp30, CD16 and 2B4, and upregulated their inhibitory receptors NKG2A and PD-1 compared with NK cells from the blood of the same patients [254]. A significantly increased number of Tregs in peripheral blood, lymph nodes and tumors in HNSCC patients have been observed [255–258], which has been correlated with cancer recurrence [259]. Although HNSCC patients show increased levels of Tregs compared to healthy controls, various studies provide conflicting results in terms of the prognostic significance of Tregs [260–262]. In addition, Tregs are increased in HNSCC patients after treatment [263]. HNSCC displays a high abundance of circulating MDSCs, which correlates with advanced stages of HNSCC [264]. Although the main function of MDSCs is the inhibition of T cell activation, a study by Zheng et al. demonstrated that MDSCs-derived caspase-1 promotes HNSCC cancer cell proliferation in a T cell-independent manner both in vitro and in vivo [265]. Moreover, several studies demonstrated that targeting MDSCs leads to enhanced antitumor immunity via increasing the number of CD8+ cytotoxic T cells in HNSCC [266–268].

Immunohistochemical analyses of primary OSCC report higher density of CAFs in over 60% cases, while healthy tissues and adjacent stroma of premalignant lesions show no staining [269–271]. It has been observed that increased numbers of CAFs within the primary tumor correlate with worse prognosis of HNSCC patients [272–275]. Several studies have shown that CAFs reside in the vicinity of tumor cells, thus the reciprocal interaction between CAFs and cancer cells has been suggested as the main force driving tumor development. Coculture systems of CAFs and HNSCC cancer cells revealed that tumor-CAFs crosstalk enhances the production of various tumor-promoting cytokines, chemokines, components of ECM, growth factors and MMPs. Jung et al. demonstrated that OSCC cancer cells induced upregulation of several molecules in CAFs after coculture, such as CCL7, CXCL1, CXCL2, CXCL3 and IL-8 [17]. A recent study by Álvarez-Teijeiro et al. identified several proteins differentially secreted in CAF-conditioned medium compared to normal fibroblasts, including EGF containing fibulin-like extracellular matrix protein 1 (EFEMP1), platelet derived growth factor D (PDGFD) and insulin-like growth factor binding proteins 5/7 (IBP5/IBP7) that may be responsible for sustaining the cancer stem cell phenotype in HNSCC [276]. Several studies found that HNSCC-derived CAFs express elevated levels of various molecules, such as TGF-β [277], HGF [278] and MMPs [279] compared to normal fibroblasts. Takahashi et al. demonstrated that, compared to normal fibroblasts, CAFs suppressed T cell proliferation and induced T cell apoptosis and the differentiation of PBMCs into Tregs more efficiently, which suggests an important role of HNSCC-derived CAFs in immunosuppression. Their results also showed an increased expression of IL-6, CXCL8, TNF, TGFB1, and VEGFA in CAFs compared to normal fibroblasts [280]. Bagordakis et al. identified number of overexpressed proteins related to ECM organization, ECM disassembly and metabolic processing of collagen in the CAFs secretome compared to normal oral fibroblasts, such as fibronectin type III domain-containing protein 1 (FNDC1), serpin peptidase inhibitor type 1 (SERPINE1) and stanniocalcin 2 (STC2) [281].

It is well known that ECM plays a crucial role in HNSCC development. Reportedly, the major ECM proteins involved in HNSCC development and progression are collagen, laminin and fibronectin [282]. Immunohistological studies of different histological grades of HNSCC show that distribution of ECM proteins, such as collagen and laminin, decrease depending on increased grade [283–285]. Harada et al. found that decreased expression of laminin, collagen type IV and vitronectin, and increased expression of fibronectin and tenascin correlated with the invasive phenotype of primary OSCC tumors [286]. In addition, an immunohistochemical analysis by Fabricius et al. investigated the expression of integrins αvβ3, αvβ5, α5β1 and their ligands osteopontin, vitronectin, fibronectin and fibrinogen in primary HNSCC tissues. Their results suggest that interactions αvβ3-osteopontin, αvβ3-fibronectin and α5β1-fibronectin play a role in HNSCC angiogenesis and interactions α5β1-fibronectin and αvβ5-vitronectin in HNSCC cancer cell behavior [287].

#### Epithelial-mesenchymal transition

**Fig. 2:**
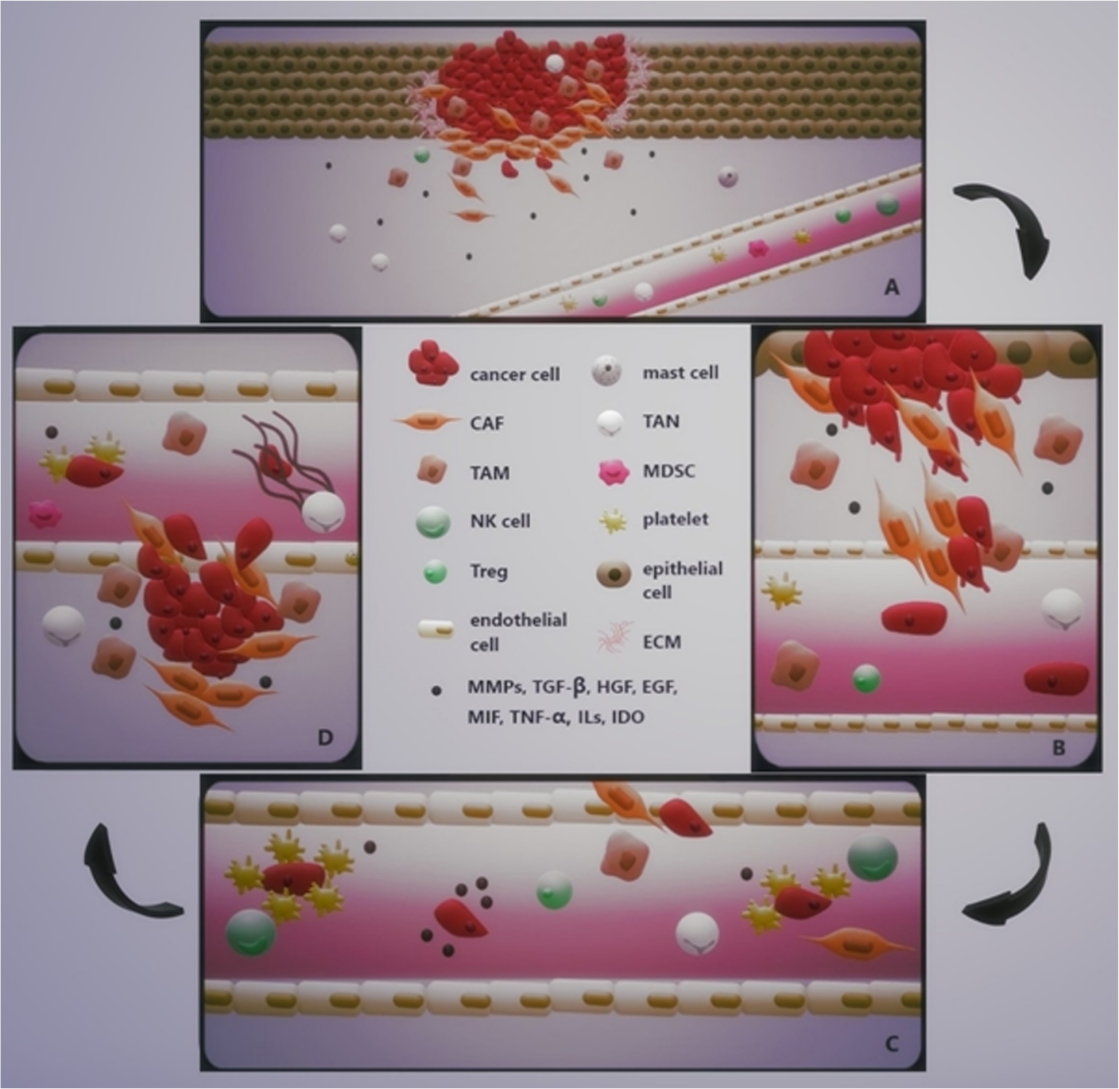
Metastatic cascade. **a** Acquisition of metastatic potential via epithelial-mesenchymal transition, degradation of the ECM (secretion of MMPs) and invasion through the basement membrane. Immune cells are recruited to the primary tumor site via cancer cell-derived and CAF-derived factors and cytokines. **b** Intravasation of cancer cells via invadopodia formation. Cancer cells acquire the resistance to anoikis. **c** Survival in the circulation. Cancer cells mediate the so-called tumor cell-induced platelet aggregation (TCIPA) to form a “platelet cloak” in order to be protected from TNF-α and to escape NK cells. Cancer cells evade the immune system by upregulation of indoleamine 2,3-dioxygenase (IDO). **d** Extravasation and formation of a secondary tumor site. Arrest of tumor cells on the endothelium, sequestration of tumor cells via NET formation, followed by transendothelial migration and invasion into the surrounding tissue. Abbreviations: TAM tumor-associated macrophage, TAN tumor-associated neutrophil, MDSC myeloid-derived suppressor cell, NK natural killer cell, Treg regulatory T cell, CAF cancer-associated fibroblast, ECM extracellular matrix, MMPs matrix metalloproteinases, MIF migration inhibitory factor, TGF-β transforming growth factor-β, EGF epithelial growth factor, HGF hepapocyte growth factor, TNF- α tumor necrosis factor-α, IDO indoleamine 2,3-dioxygenase, NET neutrophil extracellular trap

Epithelial-mesenchymal transition (EMT) is a dynamic process in cancer development, during which polarized epithelial tumor cells acquire a mesenchymal phenotype. This shift to a mesenchymal phenotype is characterized by the loss of cell adhesion and upregulation of various components of the extracellular matrix, followed by increased migratory potential and enhanced invasiveness (shown in Fig. 2). EMT is associated with the loss of proteins involved in cell junctions, such as E-cadherin and β-catenin, and with an upregulated expression of mesenchymal markers such as α-SMA, vimentin, FSP-1 and N-cadherin [288–290]. The loss of E-cadherin and high vimentin levels have been associated with tumor progression and an increase of metastases in HNSCC patients [291].

In order for tumor cells to migrate to local and distant sites, tumor and the surrounding stroma cells acquire the ability to proteolytically degrade the basement membrane and underlying collagen matrix. This degradation of, and invasion through, ECM largely depends on the function of filament-like protrusions formed on invading tumor cells, termed invadopodia, and many recent studies suggest a crucial involvement of invadopodia-mediated ECM remodeling during EMT. These structures contain various proteins such as actin regulators cortactin, dynamin and neural Wiskott–Aldrich syndrome protein (N-WASP) [292]; adhesion proteins including many integrins [293]; adaptor proteins Tyr kinase substrate with four SH3 domains (TKS4) and Tyr kinase substrate with five SH3 domains (TKS5) [294]; and many MMPs such as MT1-MMP and MMP-2 [295]. It has been observed many types of cancer cells, including HNSCC, form invadopodia, which has been correlated to their invasive phenotype in vitro and in vivo [296–300]. Invadopodia facilitate the ECM degradation in a variety of cancers through the regulation of various MMPs, primarily MMP-14 (also known as MT1-MMP), MMP-2 and MMP-9 [301, 302]. MMPs commonly overexpressed in HNSCC include MMP-1, MMP-2, MMP-3, MMP-7, MMP-8, MMP-9, MMP-10, MMP-11, MMP-13, and MT1-MMP. The expression of secreted MMP-1, MMP-2, MMP-9 and transmembrane protease membrane type 1 MMP are commonly associated with HNSCC progression. MMP-2 and MMP-9 levels have been reported in correlation with local invasion, cervical nodal metastasis, tumor progression and prognosis of HNSCC patients. In addition, high levels of MMP-9 have been detected at the invasive tumor front (ITF), thus many studies describe MMP-9 as a potential marker of invasive OSCC [303–305]. MT1-MMP, which is involved in the regulation of MMP-2 activity, has been considered a crucial protease in HNSCC, since its expression is dysregulated in 75% to 100% of HNSCC tumors. The activity of MMPs is regulated by tissue inhibitors of metalloproteases (TIMPs) [306], secreted mainly by fibroblasts in the stroma. These molecules serve as inhibitors of the catalytic activity of MMPs, as well as activators of pro-MMPs, the latter represented by TIMP-2 required for activation of pro-MMP-2. Among the most commonly identified TIMPs in HNSCC have been TIMP-1 and TIMP-2. Upregulated levels of TIMP-1 expression have been associated with poor survival, while levels of TIMP-2 have been often reported to be unchanged between HNSCC tumors and adjacent tissue. Regarding the invasion and migration of cancer cells, invadopodia formation and secretion of MMPs, the overexpression of neural precursor cell expressed developmentally downregulated 9 (NEDD9) has been suggested as a biomarker of tumor agressiveness in many types of cancer, including oral cancer. Lucas et al. demonstrated that VEGF-stimulated HNSCC cell migration and invasion was NEDD9-dependent, while immunohistochemical analysis revealed that NEDD9 co-localized to invadopodia with MT1-MMP [307]. Their following studies investigated the role of NEDD9 in secretion of MMPs, MMP-9 and MMP-2 in particular, the formation of invadopodia, as well as the interactions of NEDD9 with vimentin and non-muscle myosin IIA [308, 309]. Consistent with their findings, the high-throughput gene expression profiling of HNSCC tumor samples has shown that overexpression of NEDD9 is associated with invasive HNSCC [310]. Recent studies examined the potential involvement of stromal cells on the invadopodia formation and EMT induction in HNSCC. A study conducted by Gao et al. demonstrated that HNSCC cells were able to recruit and educate monocytes into M2 macrophages in a co-culture system via the CCL2/CCR2 axis, and these M2 macrophages then enhanced the invadopodia formation, thus invasion and migration of HNSCC cells. This study also implicated macrophages to be crucial for the induction of EMT in HNSCC cells, since the majority of macrophages have been detected at the leading front of the scratch during the wound healing assay [311]. In a follow-up study Gao et al. implicated that upregulated levels of EGF and TGF-β secreted by TAMs in direct and indirect co-culture systems with HNSCC cells induce EMT of HNSCC cells via activation of the EGFR/ERK1/2 signaling pathway [312]. Another study investigated the role of M1 and M2 macrophages in EMT induction in a co-culture system with tongue carcinoma cells, in which they showed that the interaction between cancer cells and M2 macrophages induces migration and invasion in 3D model. Macrophages as well as cancer cells exhibited altered secretome, such as upregulated expression of TGF-β, EGF and M-CSF [313]. In contrast, a study by Smirnova et al. showed that although macrophages invade together with tumor cells in vivo, the invasion of HNSCC cells was not macrophage-dependent [314]. TAMs produce macrophage migration inhibitory factor (MIF), which has been associated with EMT in many types of cancer including HNSCC. Zheng et al. demonstrated that knock-down of MIF inhibited proliferation and migration of OSCC cells [315]. Another study showed that neutrophils can be recruited by HNSCC-derived MIF via a CXCR2 mechanism in vitro. In addition, MIF promoted invasive phenotype of HNSCC cells via neutrophil-secreted CCL4 and MMP9 [316]. Trellakis et al. observed that neutrophils from HNSCC patients displayed reduced apoptosis compared with healthy donors, which has been associated with upregulated secretion of HNSCC-derived MIF [317]. Furthermore, neutrophils have been linked to the invadopodia formation in HNSCC cancer cells. Glogauer et al. demonstrated that a co-culture system of neutrophils and OSCC cancer cells increased the invasiveness of OSCC, invadopodia formation and matrix degradation through increased secretion of TNF-α and IL-8 in a contact-independent manner [318]. Also, a study conducted by Dumitru et al. has shown that neutrophils promote migration of HNSCC by increasing cortactin phosphorylation in cancer cells in vitro [319]. The role of MDSCs in EMT induction of HNSCC has not yet been extensively studied. However, being a major source of MMP-9, EGF, bFGF and TGF-β, MDSCs have been heavily implicated with the EMT promotion and neoangiogenesis in several other types of cancer [320–323]. Furthermore, there is increasing evidence MDSCs may play a crucial role in establishing the pre-metastatic niche. The exact mechanism of the pre-metastatic niche formation has not yet been fully described, however, it has been suggested the microenvironment of the distant organ site can be altered by the primary tumor itself prior to tumor cell dissemination. Primary tumor cells promote the formation of supportive metastatic microenvironment via secretion of various cytokines and growth factors, such as VEGF, placental growth factor (PlGF), TGF-β and TNF-α, granulocyte-colony forming factor (G-CSF), versican and lysyl oxidase (LOX) into the circulation to mobilize and recruit other supporting cells that interact with stromal cells and ECM of the secondary site, thus establishing the microenvironment suitable for the formation of metastases [324]. Sceneay at al. suggested that tumor-derived monocyte chemoattractant protein-1 (MCP-1) regulates the accumulation of MDSC in the pre-metastatic niche. In addition, although also the number the NK cells in the pre-metastatic niche was increased, their cytotoxic effector function was compromised, which resulted in metastasis formation [325]. Another study conducted by Wang et al. demonstrated that VEGFA secreted by cancer cells stimulates TAMs to produce CXCL1, which results in the recruitment of MDSCs to form the pre-metastatic niche [326]. Shi et al. reported that mo-MDSCs accumulate in the lungs of tumor-bearing mice before the arrival of tumor cells and that these cells secrete IL-1β to stimulate expression of E-selectin, which results in metastasis formation [327]. The mechanism of pre-metastatic niche formation in HNSCC, however, has not yet been extensively investigated. It has been demonstrated that MDSCs, as well as neutrophils and macrophages, can be recruited to the tumor site via inflammatory protein calprotectin (S100A8/A9; MRP8/14) [328–330]. During inflammation, calprotectin is actively secreted by many types of cells in the microenvironment, such as neutrophils, macrophages, monocytes and MDSCs to modulate the inflammatory response by pro-inflammatory cytokine secretion, reactive oxygen species (ROS) and nitric oxide (NO) [331–333]. The role of calprotectin in EMT has not yet been fully elucidated; however, it has been implicated in the promotion of metastatic spread by MDSCs [334]. It has been reported, calprotectin activates the MAPK and NF-κB signaling in cancer cells, thus promoting metastasis [335–337] and is strongly upregulated in several types of cancer [338]. However, the levels of expression of calprotectin in primary HNSCC are downregulated compared with other types of cancer [339–342]. Silva et al. reported, that in HNSCC calprotectin contributes to the regulation of MMP-2 expression and secretion in the 3D cell culture, thus inhibiting invasion and migration of cancer cells [343].

Representing the most abundant cell type within the tumor microenvironment, the role of CAFs in the process of EMT in many types of cancer, including HNSCC, has been intensely researched. Many studies show that the presence of CAFs promotes cancer cell invasion [22, 344–349]. It has been reported CAFs enhance the invasion of cancer cells via various mechanisms, such as MMP-mediated ECM degradation and subsequent release of latent growth factors [22]; matrix stiffening through integrin-mediated mechanotransduction and through actomyosin contractility [150, 350]; secretion of soluble factors, including HGF and TGF-β [345, 351, 352]; secretion of exosomes [55]; and direct cell-cell contact [353]. The stimulating effect of CAFs on HNSCC invasion has been described by various in vitro assays [354–356]. The possible contribution of CAFs to the EMT induction in HNSCC carcinoma cells has been implicated by immunohistochemical analyses, in which markers associated with EMT in CAFs in paired primary and metastatic OSCC showed that Ki-67+ metastatic carcinoma cells downregulate E-cadherin when in direct contact with CAFs [357]. In addition, various in vitro studies demonstrated that EMT in HNSCC cells can be induced by CAF-derived molecules, such as SDF-1 via activation of the PI3K-Akt/PKB signaling pathway [358], TGF-β1 via the TGF-β/Smad signaling pathway [359], endothelin-1 [360] and CCL-7 [17]. Richter et al. demonstrated that TGFβ1/ EGF long-term co-stimulation enhances the invasive phenotype of OSCC, such as significantly upregulated expression of MMP-2 and MMP-9, compared with single growth factor stimulation [361]. A study conducted by Wu et al. examined the effect of Gal-1 on OSCC cell invasion and migration. It has been observed that blocking Gal-1 expression inhibits cancer cell migration and invasion induced by CAF-conditioned medium via MCP-1/CCR2 signaling pathway. Furthermore, in vivo study revealed that Gal-1 knockdown in CAFs efficiently inhibits metastasis in vivo [362]. Knowles et al. reported that HNSCC-derived CAFs contribute to the HNSCC invasion and metastasis via activation of the HGF/c-Met signaling axis in vitro [363]. Their following study showed the effects of CAFs on HNSCC metastasis in a mouse model. The co-injection of CAFs with HNSCC cells resulted in increased tumor growth, disease spread to the lymph nodes and lung metastases when compared to the injection of HNSCC cells alone [364]. Several studies also report that IL-1 secretion of OSCC cells stimulates TGF-β and HGF production by CAFs, which promotes invasion of cancer cells in vitro [365, 366]. In addition, Lewis et al. show that cancer cell-derived TGF-β1 directly induced the activated phenotype in CAF, which in turn stimulate the OSCC invasion via the HGF production [367].

Beside the stromal components of tumor environment, it is reasonable to assume that also hypoxia, a crucial hallmark of cancer, may play a major role in the formation of invadopodia, in the induction of EMT and in promotion of migration and invasion of cancer cells. It has been reported that expressions of EMT promoters, Snail, Slug, TWIST and SMAD nuclear interacting protein-1(SNIP1), which are regulated by HIF-1α, correlate with induction of EMT phenotype in OSCC cells in vitro [368–370]. A study by Huang et al. reported that SLUG regulated the expression of MT4-MMP under hypoxia, which promoted the invasiveness of HNSCC cell lines [371]. Yang et al. demonstrated that hypoxia-induced TWIST activated BMI1 expression and a knock down of TWIST reversed the EMT and invasive phenotype in HNSCC under hypoxia in vitro [372]. It has been suggested that hypoxia induces EMT in OSCC via activation of the Notch signaling pathway and the inhibition of the Notch signaling pathway suppresses EMT [373]. These results are consistent with a study by Diaz et al. showing that hypoxia potentiates the invadopodia formation and ECM degradation in HNSCC in a HIF-1α-dependent manner. Furthermore, their results also implicate that the invasive phenotype of cancer cells is regulated by cell contact-dependent hypoxia-mediated Notch signaling coupled with the paracrine activation of the EGFR, which is mediated by the ADAM12-dependent secretion of HB-EGF [374]. A recent study suggests that hypoxic conditions promote EMT, metastasis and glycolysis in HNSCC via positive feedback loop between metadherin (MTDH) and HIF-1α. The study showed that hypoxia increased the expression levels of genes associated with glycolysis, such as MCT1, MCT4, GLUT1 and LDHA in HNSCC cells and stimulated uptake of glucose, production of lactate and cell invasion in vitro [375]. Several studies suggest that targeting the pathways associated with altered tumor metabolism impairs EMT, migration and invasion of HNSCC. A recent study by Li et al. demonstrated that blockage of glycolysis via targeting PFKFB3 suppressed the migration and invasion of HNSCC cells by inhibiting the invadopodia formation of HNSCC cancer cells in vitro and in vivo [376]. A study by Xu et al. showed that blockage of glycolysis by 2-DG reversed EGF-induced EMT in OSCC in vitro and moreover, the treatment of 2-DG reduced the metastatic spread to regional lymph nodes in vivo [377]. A report by Wang et al. indicates that HNSCC cell invasion and glucose metabolism is regulated via the transcription factor tripartite motif containing 24 (TRIM24)-mediated GLUT3 induction [378]. Similar results were shown in a study by Chang et al. which provided evidence that the HNSCC cell migration and invasion are regulated by the activation of the GLUT4-TRIM24 axis [379].

#### Survival in the circulation

Normal epithelial cells require direct contact with the basement membrane via integrins in order to survive and proliferate. When normal cells lose contact with the surrounding ECM or other neighboring cells, these cells undergo programmed cell death, termed anoikis, to reduce the development of metastases. However, in the case of a metastatic cascade, to develop a resistance to anoikis is a crucial step for tumor cells to disseminate from the primary tumor, survive in the circulation in an adhesion-independent manner, travel to the secondary site, extravasate and form metastases. A study by Neiva et al. described that a crosstalk between tumor-associated endothelial cells and tumor cells protected the tumor cells from anoikis. Their results demonstrated that endothelial cell-secreted factors IL-6, IL-8 and EGF induced the activation of the STAT3/Akt/ERK signaling pathways in HNSCC cells in a contact-independent manner, which lead to increased tumor cell survival and migration [380]. Several studies examined the role of the pro-survival signaling pathway c-Met/Akt in anoikis in HNSCC. It has been reported that CAF-derived HGF activated the c-Met/Akt pathway in HNSCC cells in vitro [363]. The effect of HGF on anchorage-independent tumor cell survival has been investigated in a study by Zheng et al., which showed that HGF-induced anoikis resistance was dependent on ERK and Akt pathways and the blockage of either pathway resulted in apoptosis of tumor cells. Furthermore, it has been reported the HGF-induced anoikis was independent of NFκB [381]. Their following study revealed that COX-2 provided resistance to HGF-induced anoikis in HNSCC via the activation of activator protein-1 (AP-1) through the ERK signaling pathway [382]. The neurotrophic tyrosine kinase receptor B (TrkB), which is frequently overexpressed in many cancer types including HNSCC, has been suggested as one of the major inducers of anoikis resistance [383–385]. A study by Jiffar et al. revealed that CAFs contribute to the invasive OSCC phenotype via brain-derived neurotrophic factor (BDNF)-mediated TrkB signaling axis cascade, which has been then further supported in vivo [386]. Also, the ECM proteins including collagen, fibronectin and laminin, which are major regulators of tumor cell differentiation, invasion, migration and survival, have been implicated in promoting the anoikis resistance [387]. Among matrix proteins collagen type I is the most effective in delaying anoikis in cancer cells [388]. A study by Koontongkaew et al. showed that metastatic cells plated on collagen I gel significantly upregulated their cytokine secretion, which activated MMP-2 and MMP-9 and enhanced HNSCC cell invasion [389]. Fibronectin has been also implicated in playing a role in anoikis resistance in HNSCC. Zhang et al. found that OSCC cells escape p53-induced anoikis by forming multicellular aggregates followed by integrin αv-mediated upregulation of fibronectin [390]. Their following study demonstrated that alternatively spliced V region and function-perturbing point mutations in the high-affinity heparin-binding domain of fibronectin induce anoikis in OSCC via integrin αv-mediated phosphorylation of FAK and ERK [391].

After entering the circulation, tumor cells exploit many mechanisms of immunoevasion. Numerous studies show that cancer cells acquire the ability to aggregate platelets in order to survive in the circulation, a process known as tumor cell-induced platelet aggregation (TCIPA). The formation of this “platelet cloak” provides many advantages to tumor cells, such as a shield which enables tumor cells to evade the immune systems, since platelets protect tumor cells from TNF-α [392] and NK-mediated cytotoxicity [393]; an increased extravasation of the tumor cells by the adhesion to the vascular endothelium [394]; a protection from high shear forces in the bloodstream; and a secretion of various growth factors for tumor cells to utilize [395]. Reportedly, the “platelet cloak” can also transfer platelet-derived normal MHC class I onto the tumor cell surface to help escape the T cell-mediated immunity [396]. The mechanism by which tumor cells activate platelets to form TCIPA includes the stimulation of the release of various molecules, such as ADP, MMP-2 and PGE2, and generation of thromboxane A2 (TXA_2_). This process is often stimulated by tumor cell-derived proteases, such as thrombin, cathepsin B, cancer procoagulant (EC 3.4.22.26), MMP-2 and MMP-14 [397, 398]. The interaction leads to the activation of major platelet adhesion molecules, such as integrin receptors GPIb-IX-V and GPIIb/IIIa, P-selectin and Toll-like receptor 4 (TLR4) [351, 399, 400]. Huang et al. demonstrated increased platelet aggregation in HNSCC patients, which was correlated with the tumor stage [401]. Although many studies investigated the role of the tumor cell-induced platelet aggregation in various types of cancer, the contribution of platelet aggregation to the process of immunoevasion in HNSCC has not yet been studied. Another suggested mechanism by which cancer cells survive in the circulation and evade the immune system is by upregulation of indoleamine 2,3-dioxygenase (IDO), a tryptophan-catabolising enzyme. Studies report the upregulation of IDO correlates with metastasis and worse prognosis in various types of cancers including OSCC [402]. The increased expression has been correlated with decreased numbers of CD3+ infiltrating T cells and with an upregulation of Tregs [403, 404]. Moreover, various studies report that iNOS production by peripheral blood neutrophils is significantly reduced in OSCC patients and depends on tumor stage [405, 406].

#### Extravasation

After the successful arrival at the secondary metastatic site, it is crucial for tumor cells to escape the hostile intravascular environment and extravasate into the tissue. The predominant mechanism of extravasation involves the arrest of tumor cells on the endothelium, which is followed by transendothelial migration (TEM) and invasion into the surrounding tissue. This process is characterized by alterations in endothelial cell-cell junctions. In vitro studies show, that the attachment of tumor cells onto the luminal side of the endothelial cell [407] is enabled by various adhesion ligands and receptors, such as selectins, intergrins, cadherins, immunoglobulins and CD44 [408]. However, the exact mechanism in vivo has yet to be elucidated. Using transgenic zebrafish that uniformly express GFP throughout their vasculature, Stoletov et al. confirmed the extravasation cascade and further demonstrated, that this process is mediated by Twist, VEGFA and integrin β (ITGB1) expression [409].

Besides the interaction between tumor cells and endothelial cells (ECs), the tumor-promoting immune cells also assist in successful extravasation. Suggested mechanisms, by which platelets promote extravasation and transendothelial migration, include induction of the EMT and invasiveness in tumor cells via TGF-β-mediated activation of Smad and NF-κB signaling pathways [410], as well as modulation of endothelial junctions and cytoskeleton via ATP secreted by platelets after tumor cell activation, which interacts with endothelial P2Y2 receptor in order to open the EC junctions [411]. Weber et al. demonstrated that platelets promote endothelial permeability and extravasation of tumor cells when activated by integrin αvβ3 expressed on tumor cells in vivo [412]. Furthermore, platelets contribute to the extravasation of tumor cells by recruitment of granulocytes via production of CXCL5 and CXCL7 [413]. Upon activation, neutrophils form NETs, which have been shown to promote extravasation by tumor cells sequestration [414] and MMP-9-mediated degradation of ECM [415]. Monocytes/macrophages can be recruited to the metastatic site by tumor cell-derived molecules. Reportedly, after the arrival into the lung (pulmonary metastases account for 66% of distant metastases in HNSCC [416]) tumor–platelet aggregates attached to ECs express tissue factor (TF) to stimulate the expression of VCAM-1 and VAP-1. These inflammatory mediators trigger the recruitment of macrophages, which then promote tumor cell survival and increase the vascular permeability, possibly by transmitting the pro-survival signals via VCAM-1 expressed on tumor cell surface [417–419]. Moreover, VEGF produced by macrophages and tumor cells has been shown to induce vascular permeability and transendothelial migration [420, 421].

Since HNSCC metastasizes primarily via the lymphatic invasion, Fennewald et al. investigated the interaction of HNSCC cancer cell and ECM components of lymph node parenchyma, such as laminin, fibronectin, vitronectin and hyaluronic acid in low fluid shear conditions. Their results show that HNSCC cell lines bound to laminin via α2β1, α3β1, and α6β1 integrins in a presence of lymphodynamic low shear stress, which resulted in activation of calcium signaling [422]. A study by Yen et al. demonstrated that integrin β1 promotes migration and transendothelial migration of OSCC cells via insulin-like growth factor (IGF)-independent insulin-like growth binding protein 3 (IGFBP3) [423]. The mechanisms of extravasation of tumor cells have been well described in many types of cancer; however, the effect of microenvironmental factors on extravasation in HNSCC has yet to be investigated.

#### Mesenchymal-epithelial transition

Mesenchymal-epithelial transition (MET), also known as mesenchymal-to-epithelial reverting transtition (MErT), describes a process, by which cancer cells revert back from the EMT-induced mesenchymal phenotype. The disseminated cancer cells undergo this process in order to adapt to the microenvironment of the secondary metastatic site to allow the colonization, as metastases recapitulate the primary tumor pathology. Although the precise mechanism of MET has not yet been elucidated, several studies highlight the importance of E-cadherin re-expression in the metastatic tissue. Several studies studied the molecular mechanisms of MET in HNSCC [424–427], however, the role of the cells within the surrounding microenvironment of the secondary metastatic site in HNSCC has not yet been investigated.

## Conclusions

The emerging evidence of crucial contribution of different stromal components to the regulation of the HNSCC development implicates a fundamental role of the tumor microenvironment in providing a supportive niche, thus substantially promoting HNSCC development and metastasis. While the research has previously focused mainly on altered expression of genes and aberrant genetic and epigenetic mutations in tumor cells, it is becoming clear that investigation of differences in stromal composition of the HNSCC tumor microenvironment and their impact on cancer development and progression may help better understand the mechanisms behind different responses to therapy, thus help define possible targets for clinical intervention.

## Abbreviations

4-NQO: 4-nitroquinoline 1-oxide
5-HT: 5-hydroxytryptamine
ADAM: A disintegrin and metalloproteinase
ADCC: Antibody-dependent cellular cytotoxicity
AP: Activator protein
BDMC: Bone marrow-derived mesenchymal cell
BDNF: Brain-derived neurotrophic factor
CAF: Cancer-associated fibroblasts
CCL: Chemokine (C-C motif) ligand;
COX: cyclooxygenase
CPA: Carboxypeptidase A
CTLA: Cytotoxic T-lymphocyte-associated protein
CXCL: Chemokine (C-X-C motif) ligand
CXCR: Chemokine (C-X-C motif) receptor
DC: Dendritic cell
EC: Endothelial cell
ECM: Extracellular matrix
EdMT: Endothelial-mesenchymal transition
EFEMP1: EGF containing fibulin-like extracellular matrix protein
EGF: Epithelial growth factor
EMT: Epithelial-mesenchymal transition
FAK: Focal adhesion kinase
FAP: Fibroblast-activation protein
FGF: Fibroblast growth factor
FNDC: Fibronectin type III domain-containing protein
FOXP3: Forkhead box P3
FSP: Fibroblast-specific protein
Gal: Galectin
GITR: Glucocorticoid-induced tumor necrosis factor receptor family-related protein
GLUT: Glucose transporter
GM-CSF: Granulocyte-macrophage colony-stimulating factor
GP: Glycoprotein
GSK: Glycogen synthase kinase
HGF: Hepatocyte growth factor
HIF: Hypoxia inducible factor
HLA-DR: Human leukocyte antigen DR isotype
HNC: Head and neck cancer
HNSCC: Head and neck squamous cell carcinoma
HPV: Human papillomavirus
IBP: Insulin-like binding protein
ICAM: Intercellular adhesion molecule
IDO: Indoleamine 2,3-dioxygenase
IFN: Interferon
IL: Interleukin
iNKT: Invariant neutral killer T cell
iNOS: Inducible nitric oxid synthase
ITF: Invasive tumor front
ITGB: Integrin beta
LAMP: Lysosomal-associated membrane protein
LDH: Lactate dehydrogease
LOX: Lysyl oxidase
LPS: Lipopolysacharide
LT: Leukotrien
MAPK: Mitogen-activated protein kinase
MC: Mast cell
MCD: Mast cell density
MCP: Monocyte chemoattractant protein
MCT: Monocarboxylate transporter
MDSC: Myeloid-derived suppressor cell
MErT: Mesenchymal-epithelial reverting transition
MET: Mesenchymal-epithelial transition
MHC: Major histocompatibility complex
MIF: Migration inhibitory factor
MMP: Matrix metalloproteinase
MMT: Mesothelial-mesenchymal transition
MTDH: Metadherin
MVD: Microvessel density
NEDD9: Neural precursor cell expressed developmentally downregulated 9
NET: Neutrophil extracellular trap
NF-κB: Nuclear factor κB
NK: Neutral killer cell
NO: Nitric oxid
OSCC: Oral squamous cell carcinoma
OXPHOS: Oxidative phosphorylation
PBMC: Peripheral blood mononuclear cell
PDGFR: Platelet-derived growth factor receptor
PD-L1: Programmed death receptor ligand 1
PFKFB3: 6-phosphofructo-2-kinase/fructose-2,6-biphosphatase 3
PG: Prostaglandin
PI3K: Phosphatidylinositol 3-kinase
PlGF: Placental growth factor
PMN: Polymorphonuclear leukocyte
PPAR: Peroxisome proliferator-activated receptor
PSGL: P-selectin glycoprotein ligand
PTEN: Phosphatase and tensin homolog
RANTES: Regulated on activation, normal T cell expressed and secreted
ROS: Reactive oxygen species
SCF: Stem-cell factor
SDF: Stromal cell-derived factor
SERPINE: Serpin peptidase inhibitor
SMA: Smooth muscle actin
STAT: Signal transducer and activator of transcription
STC: Stanniocalcin
TAM: Tumor-associated macrophage
TAMC: Tumor-associated mast cell
TAN: Tumor-associated neutrophil
TCIPA: tumor cell-induced platelet aggregation
TCR: T cell receptor
TEM: Transendothelial migration
TF: Tissue factor
TGF: Transforming growth factor
Th: Helper T cell
TIL: Tumor infiltrating leukocyte
TIMP: Tissue inhibitor of metalloproteases
TKS: Tyr kinase substrate
TLR: Toll-like receptor
TNF: Tumor necrosis factor
TRAIL: TNF-Related apoptosis-inducing ligand
Treg: Regulatory T cell
TRIM24: Tripartite motif containing 24
TrkB: Tropomyosin receptor kinase B
TXA2: Thromboxane A2
VAP: Vascular adhesion protein
VCAM: Vascular cell adhesion protein
VEGF: Vascular endothelial growth factor
VTE: Venous tromboembolism
VWF: Von Willebrand factor
YAP: Yes-associated protein
